# Burden of digestive congenital anomalies in the Middle East and North Africa region between 1990 and 2021

**DOI:** 10.1186/s41043-026-01398-4

**Published:** 2026-07-18

**Authors:** Fatemeh Akhlaghi, Azin Kadkhodamanesh, Faegheh Bahri, Reyhane Khajezade, Arya Adinepour, Mahsa Iranmanesh, Mohammad Amin Khajuei Gharaei, Zahra Sarkoohi, Mohammad-Mahdi Bastan, Hossein Pourghadamyari, Hamid Sharifi, Omid Eslami, Seyed Aria Nejadghaderi

**Affiliations:** 1 Department of Pediatric and Neonatal Intensive Care Nursing, Razi Faculty of Nursing and Midwifery, Kerman University of Medical Sciences, Kerman, Iran; 2 Student Research Committee, Razi Faculty of Nursing and Midwifery, Kerman University of Medical Sciences, Kerman, Iran; 3 HIV/STI Surveillance Research Center, and WHO Collaborating Center for HIV Surveillance, Institute for Futures Studies in Health, Kerman University of Medical Sciences, Kerman, Iran; 4 Department of Clinical Biochemistry, Afzalipour Faculty of Medicine, Kerman University of Medical Sciences, Kerman, Iran; 5 Student Research Committee, Faculty of Public Health, Kerman University of Medical Sciences, Kerman, Iran; 6 Student Research Committee, Afzalipour Faculty of Medicine, Kerman University of Medical Sciences, Kerman, Iran; 7 Applied Cellular and Molecular Research Center, Kerman University of Medical Sciences, Kerman, Iran; 8 Institute for Global Health Sciences, University of California, San Francisco, CA USA; 9 Gastroenterology and Hepatology Research Center, Institute of Basic and Clinical Physiology Sciences, Kerman University of Medical Sciences, Kerman, Iran; 10 Knowledge Hub for Migrant and Refugee Health, Institute for Futures Studies in Health, Kerman University of Medical Sciences, Kerman, Iran

**Keywords:** Digestive Congenital Anomalies, Disability-Adjusted Life Years, Prevalence, Incidence, Middle East, Sociodemographic Index, Global Burden of Disease

## Abstract

**Background:**

Digestive congenital anomalies (DCAs), the fourth leading cause of death and disability among all congenital birth defects, have a substantial impact on both the duration and quality of life. This study aimed to present the burden and socioeconomic associates of DCAs in the Middle East and North Africa (MENA) region from 1990 to 2021.

**Methods:**

DCAs were defined based on the International Classification of Diseases codes (ICD-9: 750–751.9, 756.6–756.79; ICD-10: Q38–Q45.8, Q79.0–Q79.59). Mortality was estimated using the Cause of Death Ensemble Model, and DisMod-MR 2.1 was employed to model cause-specific mortality and excess mortality. Metrics were presented as crude counts and age-standardized rates per 100,000, with 95% uncertainty intervals. The association between socio-demographic index (SDI) and DCAs burden was examined using smoothing spline models.

**Results:**

In 2021, the age-standardized incidence rate of DCAs in the MENA region was 7.17 (5.64, 9.15), while the age-standardized point prevalence was 50.57 (40.88, 59.51) per 100,000 individuals. MENA had a DALY rate of 67.85 (47.56, 88.75) and a death rate of 0.74 (0.50, 0.97). From 1990 to 2021, there were significant reductions in the age-standardized rates of incidence (-25.99% [-33.70, -17.06]), prevalence (-10.55% [-18.88, -2.04]), DALY (-62.35% [-78.36, -10.86]), and death (-62.97% [-78.95, -10.76]) associated with DCAs in MENA. In 2021, Afghanistan had the greatest age-standardized DALY rate, while Qatar had the lowest. Males generally had a greater burden of DCAs. A negative association was identified between age-standardized DALY rates and the SDI for DCAs in the MENA.

**Conclusions:**

From 1990 to 2021, we observed a significant decrease in the burden of DCAs in MENA, although the trends vary across countries. These findings offer crucial insights for developing effective prevention and management strategies for these anomalies.

## Introduction

Congenital birth defects are structural anomalies that develop during embryonic growth and can lead to significant physical and cognitive impairments or even life-threatening conditions. These defects result from a complex interplay of genetic factors (such as mutations and chromosomal anomalies), environmental influences (like maternal nutrition and exposure to drugs or infections), and epigenetic regulation [1]. Among the various types of congenital birth defects, digestive congenital anomalies (DCAs) are particularly significant, affecting the digestive tract. Common DCAs include conditions such as esophageal atresia, duodenal atresia, and anorectal malformations, which can cause severe complications such as feeding difficulties, malnutrition, and developmental delays [2]. Notably, DCAs ranked as the fourth leading cause of death and disability among all congenital anomalies [3]. These conditions have a negative impact on life expectancy and impose a substantial economic burden, with an estimated cost of $845 million, accounting for approximately 65% of total hospital expenditures for digestive system conditions in neonates. Notably, digestive system conditions ranked as the second-largest contributor to healthcare costs among all surgical conditions. DCAs, as a major subset, play a critical role in this financial burden [4]. Furthermore, managing DCAs often requires complex and prolonged hospital stays, including intensive neonatal care, which places a substantial burden on both healthcare systems and families [4].

DCAs remain a major global health concern, affecting millions of children worldwide. In 2021, approximately 2.2 million cases were reported, with an age-standardized rate of 45.1 per 100,000 in children aged ≤ 14 years. Although global prevalence slightly declined over the past three decades, mortality decreased by 35.35%. The global disease burden, measured in disability-adjusted life years (DALYs), was 4.3 million, showing a 34.96% decline over the same period. In the Middle East and North Africa (MENA) region, prevalent cases among children aged 0–14 years were higher than the global average, with 212,130 cases, though it has declined by 10.55% since 1990. In addition, mortality in the region has dropped by 63.0%, with 4,200 deaths in 2021. The regional disease burden has also decreased by 40.4% in children aged ≤ 14 years, with 55,810 DALYs, indicating improved healthcare access and neonatal management [2]. A regional study conducted in Morocco revealed that 61.0% of congenital malformation cases, including DCAs, were from rural areas, where economic conditions are poorer and healthcare access is more limited [5]. Inadequate access to prenatal and neonatal healthcare services in these regions contributes to delayed diagnosis and treatment, exacerbating health outcomes [5].

Despite the existence of studies utilizing the GBD study 2021 to assess congenital birth defects and DCAs, few studies have specifically examined the burden of DCAs in the MENA region over an extended period or explored how this burden varies across countries with different socioeconomic profiles [1–3]. In particular, the regional and country-level relationship between DCAs and socioeconomic development has remained underexplored. However, substantial variability exists across regions in terms of healthcare service quality and cultural factors. This heterogeneity significantly impacts the accuracy and comparability of disease estimates [2]. As a result, understanding and addressing the complexity of health inequalities across countries and territories have become necessary [1]. Therefore, this study utilized data from the GBD study 2021 to provide a report on the epidemiology, burden, and socioeconomic associations of DCAs in the MENA region from 1990 to 2021.

## Methods

This analysis covers DCA-related incidence, prevalence, deaths, years of life lost (YLLs), years lived with disability (YLDs), and DALYs within 21 MENA countries from 1990 to 2021, in addition to the changes within this period. We used the GBD 2021 data and framework, which provides methods for gathering, analyzing, and reporting data on 371 diseases and injuries at different global, regional, and national levels. Additional technical information about data handling and analytical procedures is available in the GBD 2021 reference materials. Previous GBD publications provide further details regarding the GBD methodology [6].

### Case definition and classification

DCAs refer to structural anomalies of the gastrointestinal tract at birth and result from disruptions in embryonic development. These anomalies, similar to other congenital disorders, are classified into four major subcategories: congenital diaphragmatic hernia, congenital malformations of the abdominal wall, congenital atresia and/or stenosis of the gastrointestinal tract, and other congenital malformations of the gastrointestinal tract. The following International Classification of Diseases (ICD) versions 9 and 10 were used for DCAs: ICD-9: 750-751.9, 756.6-756.79, and ICD-10: Q38-Q45.8, Q79.0 -79.59.

Data for DCAs burden estimation came from multiple sources, including vital registration, verbal autopsy, and surveillance systems. All sources underwent quality checks, with incomplete datasets supplemented or excluded. Missing age/sex data were imputed using GBD statistical methods [6].

### Modelling strategy

The Cause of Death Ensemble model (CODEm) was used to estimate mortality from DCAs. CODEm evaluates several statistical frameworks with varying sets of covariates and then merges the most accurate models into a cohesive estimate. The DisMod-MR 2.1 model was used to estimate cause-specific mortality for DCAs based on the Global Burden of Disease Cause of Death (GBD CoD) model and converted to excess mortality where applicable. This model incorporated random effects on prevalence and excess mortality, with a remission slope that declined with age and a maximum value of 1.0 across all ages. The excess mortality curve was smoothed to account for significant fluctuations in the excess mortality rate during the neonatal period [7].

### Statistical analysis

YLLs measure premature death by multiplying the number of deaths by the standard life expectancy at death based on the GBD reference life table. YLDs capture the impact of living with a disease, estimated by multiplying its prevalence by a disability weight, which reflects its severity. DALYs quantify disease burden by summing premature mortality (i.e., YLLs) and non-fatal health impacts (i.e., YLDs).

Metrics, including incidence, point prevalence, deaths, YLLs, YLDs, and DALYs, were presented as crude counts and age-standardized rates per 100,000 individuals. Also, 95% uncertainty intervals (UIs) were determined by running 500 statistical simulations for each estimate and taking the range between the 2.5th and 97.5th percentiles. In this study, prevalence refers to GBD point prevalence, defined as the number of existing cases at a given point in time. The association between Socio-Demographic Index (SDI) and age-standardized DALY rates was examined using smoothing spline models to capture potential non-linear trends. The SDI is a composite metric that integrates per capita income, mean years of education for individuals aged 15 and older, and the fertility rate among women under 25. It is measured on a zero to one scale, representing the spectrum from low to high socio-demographic development.

## Results

### Epidemiology of DCAs at the global, regional, and national level

#### Global level

In 2021, there were 459,018 incidence cases (95% UI: 364,141 to 585,158) and 3,216,778 prevalent cases (95% UI: 2,608,177 to 3,837,827) of DCAs globally. The age-standardized point prevalence was 45.09 per 100,000 people (95% UI: 36.64 to 54.19), indicating an 8.15% reduction from 1990 (Table 1).

**Table 1:** Prevalent cases, incident cases, deaths, DALYs, YLDs, and YLLs, along with their age-standardized rates, due to digestive congenital anomalies in 2021 for both sexes, and the percentage change in rates per 100,000 population from 1990 to 2021 in the Middle East and North Africa (MENA) region. Prevalence refers to point prevalence

	Incidence (95% UI)				Prevalence (95% UI)				DALYs (95% UI)			Deaths (95% UI)			YLDs (95% UI)			YLLs (95% UI)		
	Counts (2021)	Rate (2021)	Pcs in rate 1990–2021		Counts (2021)	Rate (2021)	Pcs in rate 1990–2021		Counts (2021)	Rate (2021)	Pcs in rate 1990–2021	Counts (2021)	Rate (2021)	Pcs in rate 1990–2021	Counts (2021)	Rate (2021)	Pcs in rate 1990–2021	Counts (2021)	Rate (2021)	Pcs in rate 1990–2021
**Global**	459018.32 (364141.41, 585158.36)	7.42 (5.89, 9.46)	-8.29 (-16.48, 1.42)		3216778.4 (2608176.62, 3837827.08)	45.09 (36.64, 54.19)	-8.15 (-14.04, -1.8)		4430696.4 (3345178.04, 5508669.1)	70.44 (53.14, 87.6)	-34.96 (-57.06, 15.48)	48483.15 (36339.69, 60374.91)	0.77 (0.58, 0.96)	-35.35 (-57.49, 16.74)	150159.86 (95738.55, 219405.66)	2.11 (1.34, 3.08)	-7.35 (-13.72, -0.8)	4280536.54 (3198479.37, 5342687.75)	68.33 (51.02, 85.34)	-35.55 (-57.68, 16.77)
**Middle East and North Africa**	41049.53 (32294.16, 52333.29)	7.17 (5.64, 9.15)	-25.99 (-33.7, -17.06)		317506.72 (255684.1, 373473.46)	50.57 (40.88, 59.51)	-10.55 (-18.88, -2.04)		394779.86 (276837.24, 516010.83)	67.85 (47.56, 88.75)	-62.35 (-78.36, -10.86)	4278.71 (2927.24, 5630.41)	0.74 (0.5, 0.97)	-62.97 (-78.95, -10.76)	14825.86 (9502.1, 21529.03)	2.37 (1.52, 3.44)	-9.67 (-18.41, 0.62)	379,954 (260082.62, 499987.74)	65.48 (44.84, 86.21)	-63.13 (-79.05, -11.03)
**Afghanistan**	4928.92 (3825.27, 6362.08)	8.48 (6.58, 10.95)	-18.13 (-32.6, -0.8)		20460.06 (16302.08, 25256.17)	45.3 (36.86, 55.48)	-15.85 (-27.28, -1.52)		73276.44 (35684.86, 129600.18)	128.82 (62.73, 227.94)	-45.43 (-68.61, 66.63)	809.62 (387.32, 1442.73)	1.43 (0.68, 2.54)	-45.63 (-68.81, 70.92)	964.28 (578.13, 1449.13)	2.12 (1.29, 3.1)	-15.41 (-33.04, 6.47)	72312.15 (34655.77, 128829.67)	126.71 (60.47, 225.97)	-45.75 (-69.01, 71.11)
**Algeria**	3433.66 (2652.26, 4452.12)	7.87 (6.08, 10.2)	-23.79 (-35.24, -9.34)		23651.49 (18886.64, 28871.13)	52 (41.58, 63.47)	-2.75 (-14.99, 13.04)		31062.62 (19897.72, 44159.1)	70.28 (45.01, 100.13)	-58.08 (-78.9, 1.09)	336.41 (210.29, 482.31)	0.76 (0.48, 1.09)	-58.81 (-79.49, 0.83)	1107.17 (704.04, 1700.91)	2.44 (1.55, 3.74)	-1.22 (-20.78, 21.41)	29955.44 (18654.26, 43052.8)	67.84 (42.22, 97.67)	-58.93 (-79.57, 1.16)
**Bahrain**	42.61 (32.91, 54.32)	5.03 (3.89, 6.41)	-38 (-47.72, -26.35)		570.03 (460.24, 704.67)	45.49 (36.82, 56.01)	-19.62 (-32.5, -6.2)		172.96 (107.84, 326.6)	18.72 (11.48, 36.05)	-70.43 (-85.56, -43.61)	1.69 (0.98, 3.42)	0.19 (0.11, 0.38)	-72.35 (-87.19, -45.17)	26.55 (16.63, 40.23)	2.13 (1.33, 3.25)	-18.9 (-35.22, 1.63)	146.41 (85.18, 296.43)	16.59 (9.63, 33.66)	-72.66 (-87.36, -45.7)
**Egypt**	6504.25 (4801.4, 8779.57)	5.26 (3.88, 7.1)	-18.05 (-31.57, -0.33)		52957.19 (41434.7, 64118.87)	44.48 (34.89, 53.38)	0.87 (-11.69, 14.33)		67592.74 (46626.21, 97321.42)	54.01 (37.3, 77.87)	-70.64 (-86.77, 17.44)	733.07 (499.03, 1062.84)	0.59 (0.4, 0.85)	-71.26 (-87.2, 18.68)	2476.8 (1544.39, 3701.4)	2.08 (1.3, 3.1)	1.86 (-17.54, 25.05)	65115.94 (44202.59, 94400.01)	51.92 (35.26, 75.29)	-71.46 (-87.31, 18.56)
**Iran**	3659.7 (2895.89, 4534.76)	7.39 (5.85, 9.16)	-34.74 (-43.48, -23.27)		37772.6 (30360.36, 44564.39)	51.84 (41.8, 61.71)	-20.14 (-28.4, -9.91)		12114.39 (7283.99, 17440.49)	22.07 (12.76, 32.48)	-87.33 (-94.98, -66.49)	119.82 (68.11, 180.8)	0.22 (0.12, 0.34)	-88.31 (-95.6, -67.72)	1735.93 (1104.06, 2549.84)	2.4 (1.54, 3.52)	-19.57 (-28.87, -8.6)	10378.46 (5780.7, 15656.15)	19.67 (10.79, 29.99)	-88.51 (-95.71, -68.07)
**Iraq**	2656.66 (2044.2, 3485.41)	6.68 (5.14, 8.76)	-34.65 (-44.23, -23.39)		19680.56 (15527.21, 23945.18)	44.6 (35.42, 54.19)	-17.77 (-28.45, -6.83)		20973.94 (13936.18, 33374.74)	51.76 (34.31, 82.49)	-55.91 (-80.47, 30.61)	225.99 (146.3, 366.16)	0.56 (0.36, 0.91)	-56.56 (-81.06, 32.68)	910.99 (550.9, 1413.54)	2.08 (1.26, 3.18)	-17.05 (-34.27, 3.07)	20062.95 (12985.13, 32489.54)	49.68 (32.18, 80.48)	-56.75 (-81.21, 32.88)
**Jordan**	1040.46 (804.83, 1370.52)	10.04 (7.77, 13.23)	-5.56 (-20.85, 14.12)		7677.47 (6056.75, 9602.51)	63.07 (50.25, 78.17)	8.15 (-8.26, 26.46)		6899.82 (4718.94, 9875.71)	65.46 (44.51, 93.92)	-51.01 (-72.94, -2.24)	73.59 (48.91, 106.35)	0.7 (0.47, 1.02)	-52.19 (-74.34, -3.23)	359.14 (222.92, 531.87)	2.97 (1.86, 4.35)	8.93 (-14.99, 39.3)	6540.69 (4347.68, 9483.49)	62.49 (41.52, 90.81)	-52.26 (-74.43, -2.85)
**Kuwait**	287.01 (219.82, 360.83)	11.98 (9.17, 15.06)	-14.92 (-29.78, 1.65)		2714.32 (2183.1, 3266.72)	78 (62.29, 94.89)	2.59 (-10.74, 18.33)		1463.35 (1118.51, 1932.42)	57.87 (43.65, 76.4)	-41.2 (-59.83, -9.37)	15.17 (11.34, 20.37)	0.61 (0.45, 0.82)	-43.04 (-61.44, -11.01)	127.55 (79.21, 185.75)	3.68 (2.28, 5.37)	3.43 (-15.75, 29.77)	1335.8 (994.4, 1795.13)	54.19 (40.19, 72.8)	-42.88 (-61.46, -9.97)
**Lebanon**	303.15 (229.79, 404.91)	7.99 (6.05, 10.67)	-13.88 (-27.7, 4.16)		2470.96 (1998.31, 2989.9)	51.89 (41.52, 62.61)	-7.63 (-21.68, 7.05)		1500.16 (980.27, 2269.16)	38.08 (24.58, 58.05)	-67.21 (-85.83, -12.2)	15.77 (9.99, 24.23)	0.4 (0.25, 0.62)	-68.4 (-86.64, -12.32)	115.16 (74.13, 174.19)	2.43 (1.57, 3.69)	-7.31 (-27.24, 17.61)	1385 (870.86, 2138.11)	35.66 (22.31, 55.29)	-68.59 (-86.78, -12.37)
**Libya**	206.8 (157.75, 272.74)	5.37 (4.1, 7.08)	-41.91 (-50.4, -32.45)		2911.83 (2366.36, 3502.03)	49.86 (40.44, 59.97)	-8.1 (-19.05, 4.27)		4089.38 (2142.96, 6986.88)	101.49 (52.53, 173.93)	-28.89 (-54.77, 13.17)	45.05 (23.17, 77.77)	1.12 (0.57, 1.94)	-28.76 (-55.14, 14.12)	135.28 (85.49, 202.64)	2.34 (1.48, 3.54)	-7.66 (-24.64, 16.56)	3954.1 (2013.42, 6837.57)	99.16 (50.29, 172.05)	-29.28 (-55.47, 13.63)
**Morocco**	1641.19 (1227.63, 2174.33)	5.36 (4.01, 7.1)	-14.64 (-27.69, 1.9)		14013.14 (11206.69, 16941.88)	39.84 (31.71, 48.19)	-4.87 (-17.8, 9.98)		7517.58 (4293.77, 15247.32)	23.99 (13.63, 48.73)	-63.51 (-85.36, -7.48)	77.31 (40.41, 166.93)	0.25 (0.13, 0.53)	-65.15 (-86.56, -7.06)	651.66 (389.66, 967.83)	1.86 (1.11, 2.77)	-4.32 (-23.67, 19.73)	6865.92 (3609.38, 14737.12)	22.14 (11.67, 47.28)	-65.31 (-86.63, -7.48)
**Oman**	248.27 (190.63, 327.44)	6.53 (5.01, 8.61)	1.31 (-15.95, 22.48)		2287.57 (1835.77, 2765.39)	51.12 (41.09, 61.81)	11.96 (-4.05, 28.2)		906.72 (500.37, 2228.11)	23.02 (12.51, 57.1)	-50.31 (-80.15, 14.98)	9.04 (4.56, 23.87)	0.23 (0.12, 0.62)	-53.09 (-83.02, 15.5)	106.48 (66.98, 160.07)	2.39 (1.5, 3.57)	13.69 (-11.41, 41.56)	800.24 (402.83, 2121.06)	20.64 (10.36, 54.71)	-53.34 (-83.21, 15.27)
**Palestine**	337.77 (260.58, 432.19)	5.85 (4.52, 7.49)	-35.02 (-46.6, -18.53)		2503.01 (1978.88, 3076.31)	42.25 (33.62, 51.56)	-20.12 (-31.71, -8.29)		1907.18 (1169.42, 3341.6)	32.64 (20.01, 57.21)	-61.97 (-83.27, -17.44)	20.16 (12.01, 36.17)	0.35 (0.21, 0.62)	-62.99 (-84.3, -16.93)	116.78 (71.26, 178.55)	1.98 (1.22, 2.98)	-19.61 (-36.7, 0.86)	1790.4 (1064.83, 3217.67)	30.66 (18.21, 55.14)	-63.22 (-84.47, -16.89)
**Qatar**	78.03 (59.71, 101.68)	4.21 (3.22, 5.48)	-33.46 (-44.36, -20.62)		1137.21 (916.78, 1352.37)	47.4 (37.77, 57.33)	-1.5 (-15.35, 16.87)		214.25 (122.64, 526.6)	10.75 (5.97, 27.21)	-66.19 (-83.75, -34.14)	1.84 (0.88, 5.44)	0.1 (0.05, 0.29)	-70.79 (-88.13, -38.87)	53.41 (34.05, 79.17)	2.23 (1.39, 3.42)	-1.25 (-22.79, 23)	160.85 (76.37, 475.67)	8.52 (4.02, 25.15)	-71.14 (-88.39, -38.85)
**Saudi Arabia**	1038.86 (776, 1385.07)	4.66 (3.48, 6.22)	-37.57 (-47.27, -27.21)		15867.22 (12838.57, 19161.95)	48.6 (38.81, 58.75)	1.09 (-9.68, 14.32)		3699.82 (1881.6, 8511.96)	14.62 (6.83, 35.47)	-81.92 (-93.93, -51.95)	36.13 (15.77, 91.07)	0.15 (0.06, 0.39)	-83.49 (-95.08, -52.79)	732.84 (452.51, 1074.79)	2.26 (1.39, 3.36)	3.03 (-15.3, 27.56)	2966.98 (1212.46, 7827.79)	12.36 (4.7, 33.52)	-84.29 (-95.57, -53.63)
**Sudan**	4493.13 (3417.5, 6103.16)	8.15 (6.2, 11.08)	-15.99 (-31.6, 10.56)		27320.18 (22019.71, 33432.46)	51.57 (41.82, 62.95)	-2.79 (-17.32, 12.94)		63614.37 (37441.68, 100991.17)	115.2 (67.87, 182.74)	-52.75 (-81.13, 97.64)	698.69 (405.05, 1121.81)	1.27 (0.73, 2.03)	-53.22 (-81.41, 104.15)	1276.2 (770.37, 1942.07)	2.41 (1.47, 3.66)	-1.69 (-22.66, 25.6)	62338.17 (36138.59, 100208.75)	112.79 (65.41, 181.25)	-53.27 (-81.4, 104.42)
**Syria**	581.51 (440.39, 776.2)	6.16 (4.66, 8.22)	-25.4 (-39.55, -5.9)		5585.53 (4434.41, 6812.61)	44.26 (34.86, 53.97)	-16.53 (-29.79, -2.34)		4286.3 (2622.46, 6982.61)	43.3 (25.94, 71.58)	-66.34 (-86.43, -2.82)	46.38 (27.42, 77.81)	0.47 (0.27, 0.79)	-66.89 (-87.12, -2.05)	257.37 (155.07, 392.77)	2.06 (1.24, 3.12)	-16.47 (-35.5, 6.46)	4028.93 (2365.92, 6785.85)	41.25 (23.99, 69.85)	-67.32 (-87.37, -2.4)
**Tunisia**	517.05 (408.7, 669.65)	6.45 (5.1, 8.35)	-37.82 (-48.41, -24.98)		4875.56 (3950, 5970.36)	47.58 (38.31, 58.21)	-17.34 (-29.35, -2.32)		3789.88 (2510.8, 5623.21)	45.6 (29.95, 67.81)	-70.17 (-86.4, -25.05)	40.44 (25.9, 62.09)	0.49 (0.31, 0.75)	-70.92 (-86.97, -24.52)	227.3 (144.06, 343.69)	2.22 (1.4, 3.37)	-16.72 (-34.17, 8)	3562.58 (2284.73, 5419.1)	43.37 (27.76, 65.79)	-71.12 (-87.03, -25.23)
**Türkiye**	5646.76 (4535.08, 7061.53)	11.65 (9.36, 14.57)	-24.65 (-38.69, -7)		52542.75 (42711.54, 63689.9)	76.4 (61.93, 92.72)	-3.41 (-19.04, 14.03)		38875.23 (27927.07, 53281.8)	76.43 (54.43, 105.8)	-75.09 (-89.64, -17.84)	413.84 (292.13, 577.18)	0.82 (0.57, 1.15)	-75.85 (-90.16, -18.51)	2500.92 (1531.92, 3702.49)	3.65 (2.24, 5.45)	-1.96 (-21.22, 23.24)	36374.31 (25495.56, 51025.87)	72.78 (50.94, 102.34)	-75.98 (-90.25, -18.64)
**United Arab Emirate**	177.34 (133.76, 232.54)	4.84 (3.65, 6.35)	-40.77 (-50.34, -29.68)		2525.04 (2024.85, 3061.58)	39.17 (30.98, 47.95)	-19.81 (-30.37, -5.72)		986.86 (521.16, 1661.71)	24.07 (12.23, 41.18)	-72.23 (-88.46, -42.21)	10.33 (4.91, 18)	0.26 (0.12, 0.45)	-73.25 (-89.36, -43.29)	117.34 (74.02, 179.5)	1.83 (1.16, 2.79)	-18.42 (-34.63, 9.18)	869.52 (415.9, 1532.19)	22.24 (10.64, 39.6)	-73.66 (-89.75, -43.54)
**Yemen**	3188.11 (2443.91, 4102.64)	6.84 (5.25, 8.81)	-8.67 (-23.04, 9.68)		17686.88 (14121.06, 21710.22)	41.46 (33.49, 50.17)	-8.35 (-20.75, 3.89)		49467.66 (26497.11, 83049.29)	106.54 (57.16, 178.66)	-45.29 (-72.15, 71.72)	544.38 (286.78, 919.17)	1.17 (0.62, 1.98)	-45.63 (-72.35, 74.95)	812.88 (502.33, 1224.11)	1.91 (1.18, 2.84)	-7.56 (-25.79, 19.45)	48654.77 (25644.15, 82130.95)	104.63 (55.14, 176.54)	-45.7 (-72.4, 75.17)

**DALY**: disability-adjusted life year; **YLD**: years lived with disability; **YLL**: years of life lost; **Pcs**: percent change; **UI**: uncertainty interval

In 2021, the global DALYs were 4,430,696 (95% UI: 3,345,178 to 5,508,669). The age-standardized DALY rate was 70.44 per 100,000 people (95% UI: 53.14 to 87.60), reflecting a 34.96% reduction since 1990. In addition, global death numbers caused by DCAs were 48,483 (95% UI: 36,340 to 60,375). The age-standardized death rate was 0.77 per 100,000 individuals (95% UI: 0.58 to 0.96), indicating a 35.35% reduction since 1990 (Table 1).

#### Regional Level: Middle East and North Africa (MENA)

In 2021, the MENA region documented 41,050 (95% UI: 32,294, 52,333) incident cases of DCAs, with an age-standardized rate of 7.17 per 100,000 (95% UI: 5.64, 9.15). This indicated a 25.99% reduction from 1990 to 2021. Also, in 2021, the MENA region documented 317,507 prevalent cases (95% UI: 255,684 to 373,473). The age-standardized point prevalence was 50.57 per 100,000 people (95% UI: 40.88 to 59.51), indicating a 10.55% reduction from 1990 (Table 1).

Furthermore, in 2021, deaths resulting from DCAs in the MENA region were 4,279 (95% UI: 2,927 to 5,630). The age-standardized mortality rate was 0.74 per 100,000 individuals (95% UI: 0.50 to 0.97), reflecting a 62.97% reduction since 1990. In 2021, the MENA region recorded 394,780 DALYs (95% UI: 276,837 to 516,011). The age-standardized DALY rate was 67.85 per 100,000 people (95% UI: 47.56 to 88.75), indicating a 62.35% reduction since 1990. In addition, the YLDs in MENA were 14,826, with a rate of 2.37 per 100,000 individuals. Moreover, YLLs caused by DCAs in MENA were 379,954, with a rate of 65.48 per 100,000 individuals, showing a 63.13% reduction since 1990 (Table 1).

#### National level: Countries within MENA

**Fig. 1:**
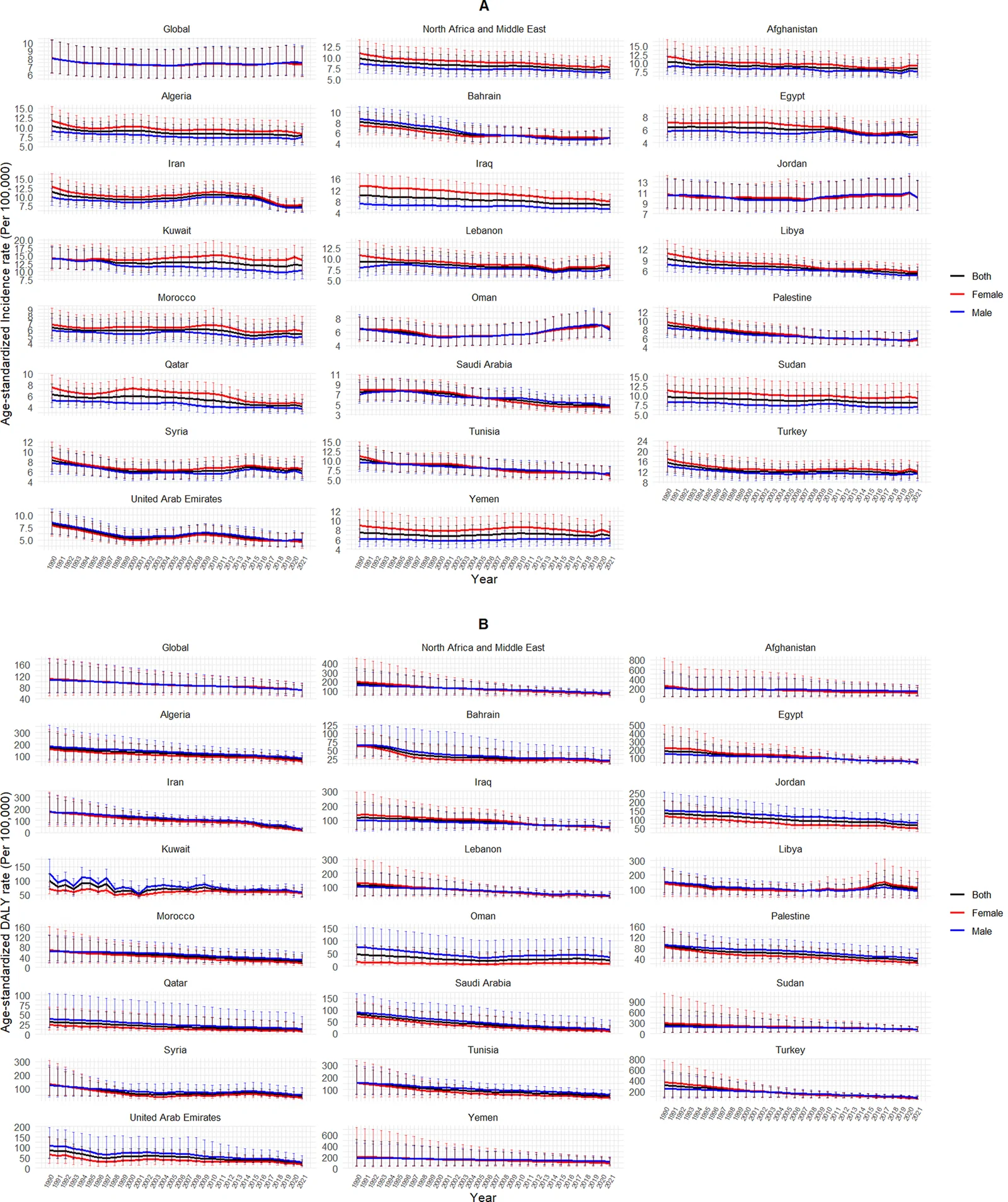

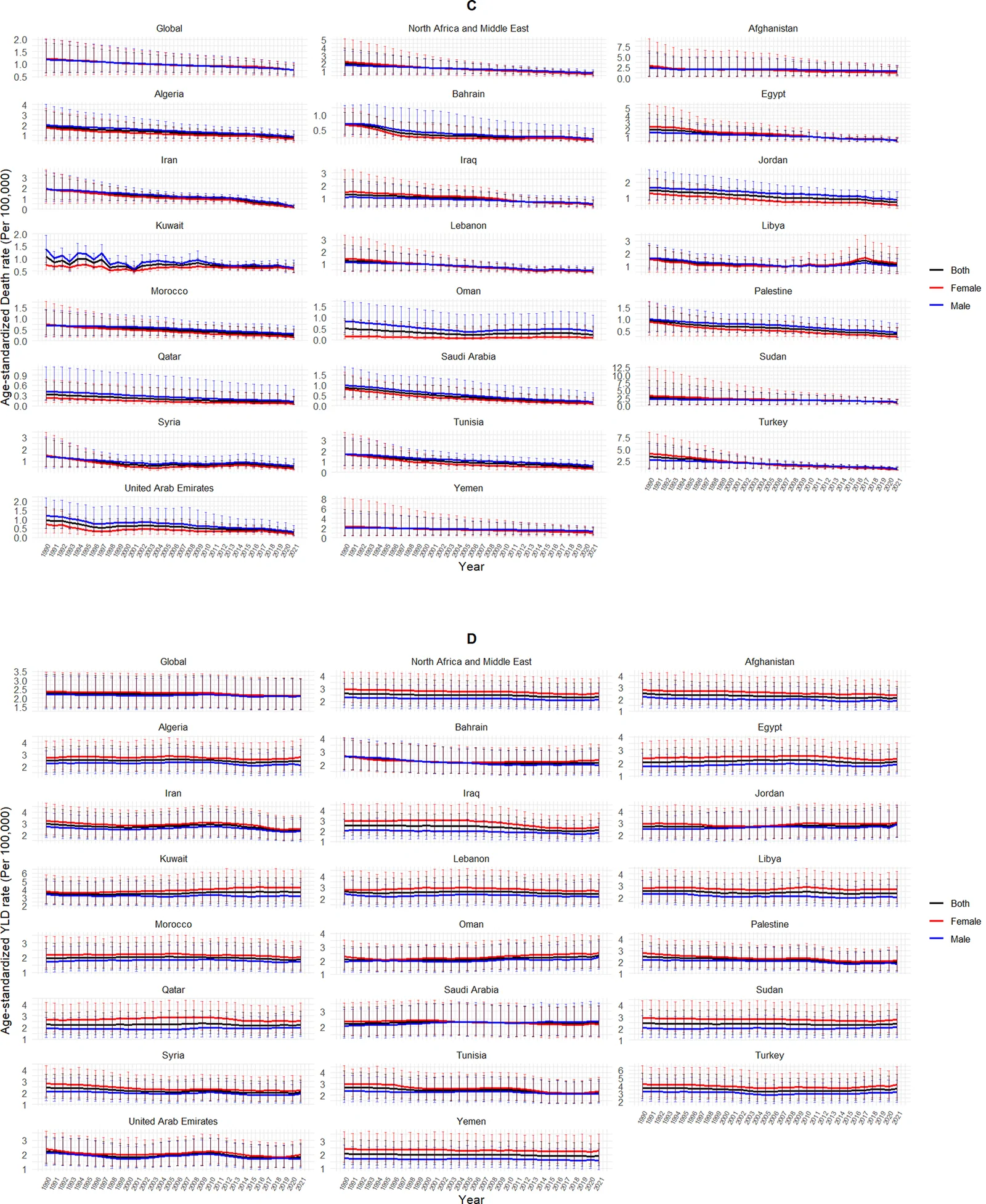

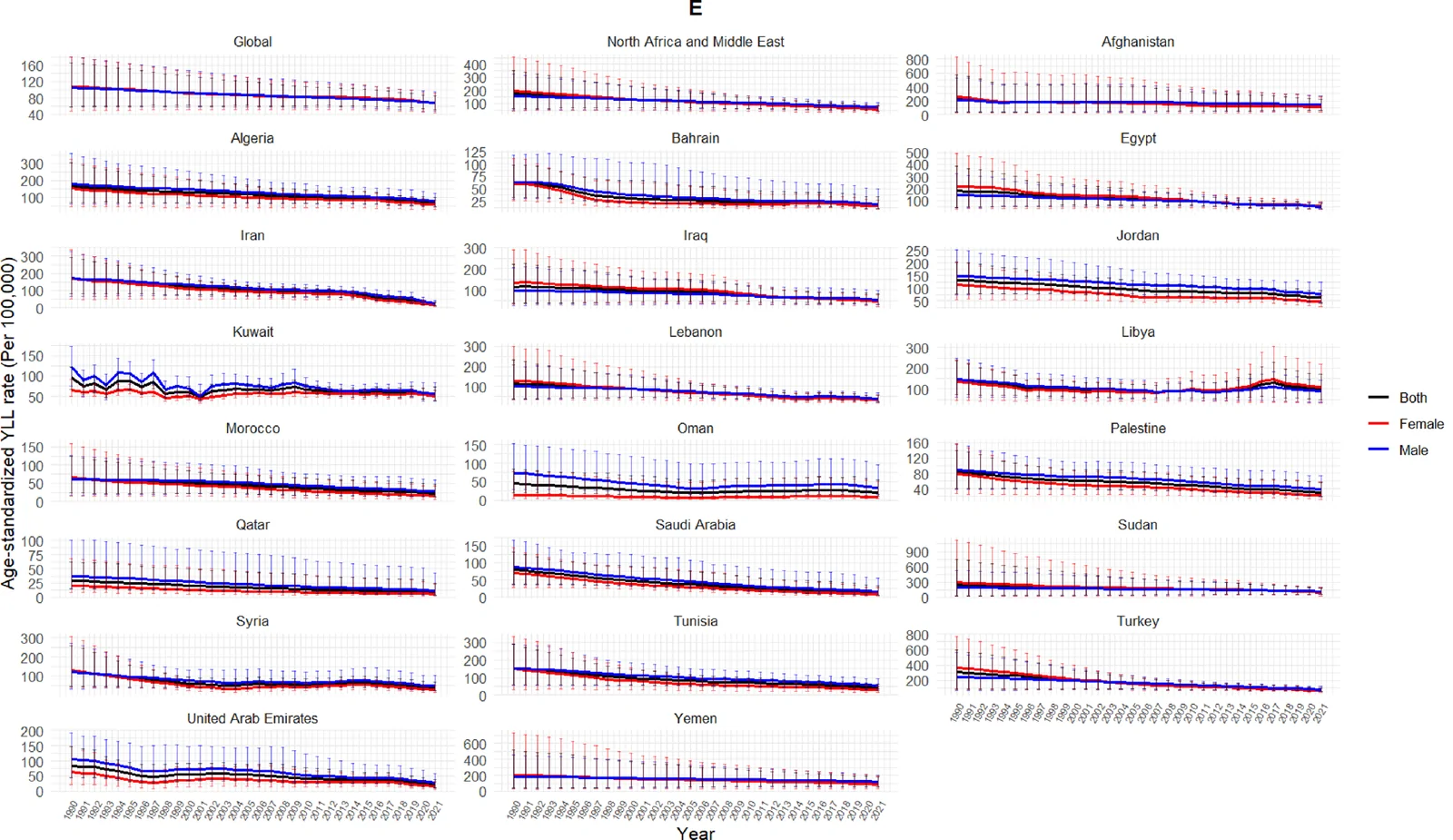
Time trends of age-standardized rates for incidence (**A**), DALYs (**B**), deaths (**C**), YLDs (**D**), and YLLs (**E**) due to digestive congenital anomalies from 1990 to 2021, categorized by location and sex. DALY: disability-adjusted life year; YLD: years lived with disability; YLL: years of life lost. (Generated from data available from http://ghdx.healthdata.org/gbd-results-tool)

Across the countries within MENA, Kuwait had the highest age-standardized incidence rate of 11.98 per 100,000 people (95% UI: 9.17 to 15.06), indicating a 14.92% decline from 1990 to 2021. However, Qatar had the lowest age-standardized incidence rate of 4.21 per 100,000 people (95% UI: 3.22 to 5.48), with a 33.46% decrease from 1990 to 2021 (Fig. 1A; Table 1).

Within MENA nations, Kuwait had 2,714 prevalent cases (95% UI: 2,183 to 3,267), with the highest age-standardized point prevalence of 78.00 per 100,000 people (95% UI: 62.29 to 94.89). In addition, the United Arab Emirates recorded 2,525 prevalent cases (95% UI: 2,025 to 3,062), resulting in the lowest age-standardized point prevalence of 39.17 per 100,000 population (95% UI: 30.98 to 47.95), indicating a 19.81% reduction from 1990 to 2021 (Table 1).

In Afghanistan, 73,276 DALYs were reported (95% UI: 35,685 to 129,600), with the greatest age-standardized DALY rate of 128.82 per 100,000 population (95% UI: 62.73 to 227.94), indicating a 45.43% reduction from 1990 to 2021. In contrast, in Qatar, there were 214 DALYs (95% UI: 122.64 to 526.60), and the lowest age-standardized DALY rate was 10.75 per 100,000 people (95% UI: 5.97 to 27.21) (Fig. 1B; Table 1).

Afghanistan reported 810 deaths (95% UI: 387 to 1,443), with the highest age-standardized death rate of 1.43 per 100,000 people (95% UI: 0.68 to 2.54), indicating a 45.63% reduction from 1990 to 2021. Furthermore, Qatar had two deaths (95% UI: 1 to 5), resulting in the lowest age-standardized death rate of 0.10 per 100,000 population (95% UI: 0.05 to 0.29), indicating a 70.79% reduction from 1990 to 2021 (Fig. 1C; Table 1).

Kuwait documented 128 YLDs (95% UI: 79 to 186), having the greatest age-standardized YLD rate of 3.68 per 100,000 population (95% UI: 2.28 to 5.37). In addition, the United Arab Emirates reported 117 YLDs (95% UI: 74 to 180), with the lowest age-standardized YLD rate of 1.83 per 100,000 population (95% UI: 1.16 to 2.79) (Fig. 1D; Table 1).

Afghanistan reported 72,312 YLLs (95% UI: 34,656 to 128,830), with the greatest age-standardized YLL rate of 126.71 per 100,000 people (95% UI: 60.47 to 225.97). Qatar documented 161 YLLs (95% UI: 76 to 476), yielding the lowest age-standardized YLL rate of 8.52 per 100,000 people (95% UI: 4.02 to 25.15) (Fig. 1E; Table 1).

### Age and sex patterns

**Fig. 2:**
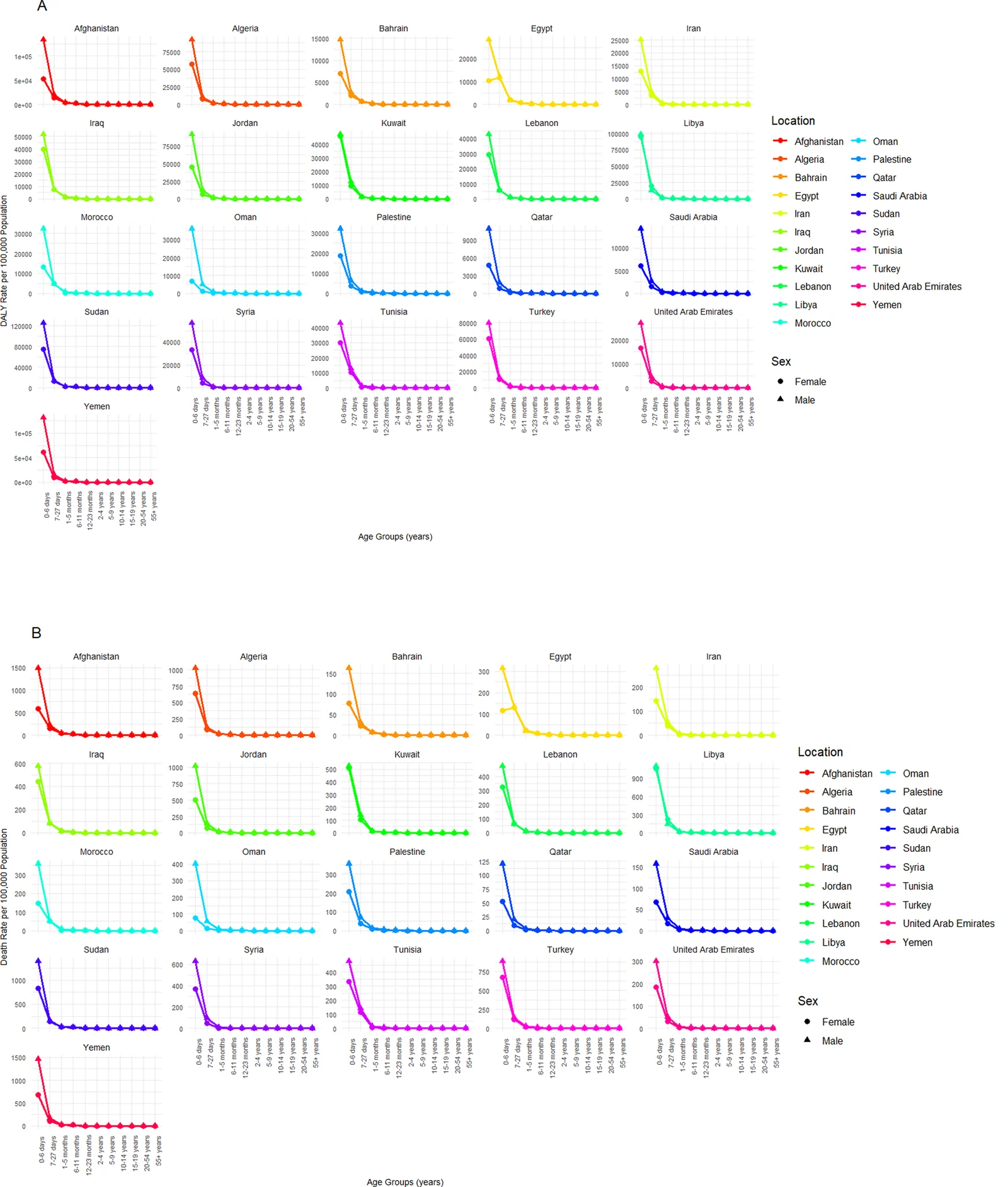

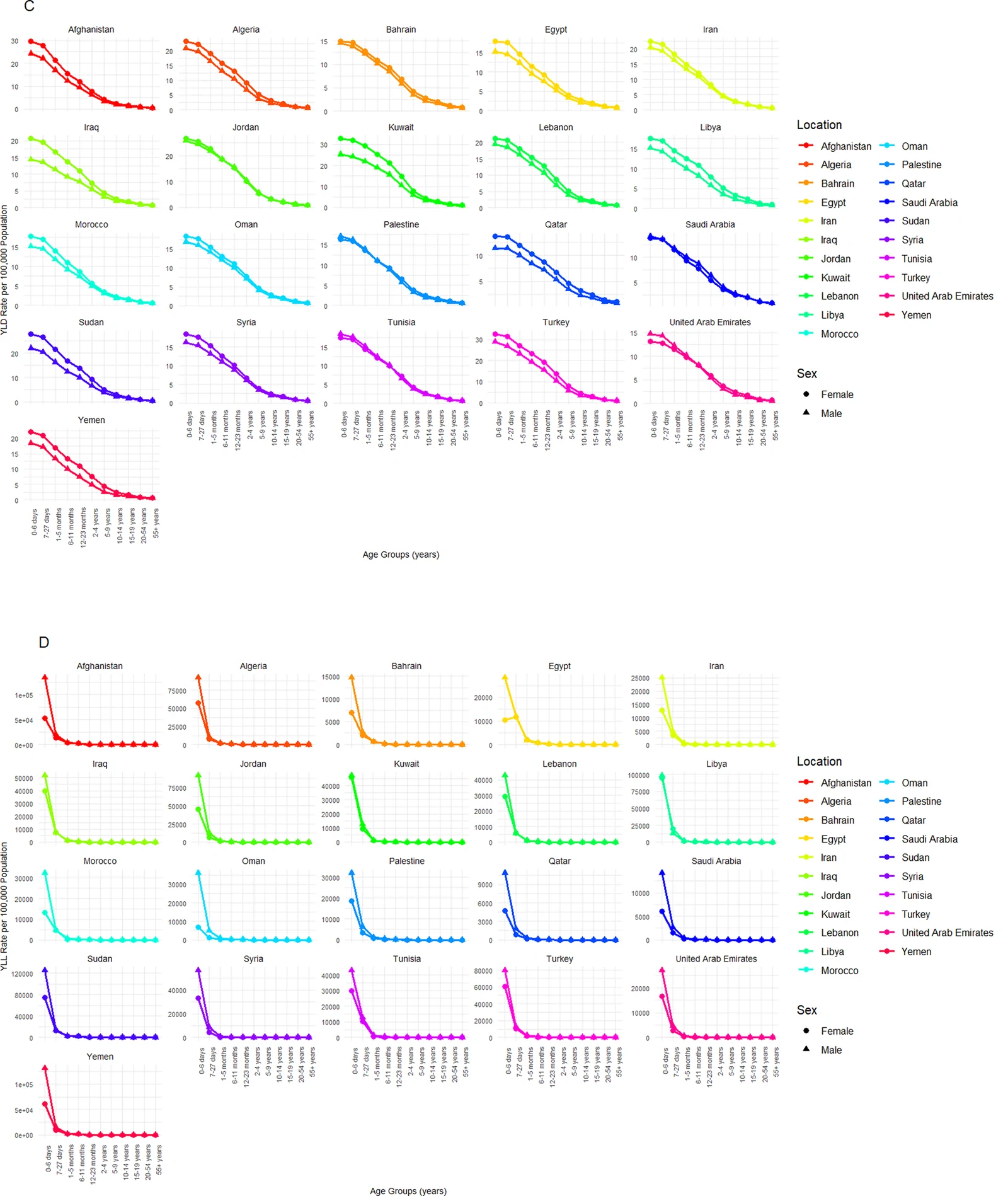
DALY rate (**A**), death rate (**B**), YLD rate (**C**), and YLL rate (**D**) for digestive congenital anomalies per 100,000 population in the Middle East and North Africa (MENA) region in 2021, categorized by location, age, and sex. DALY: disability-adjusted life year; YLD: years lived with disability; YLL: years of life lost. (Generated from data available from http://ghdx.healthdata.org/gbd-results-tool)

Those between 0 and 6 days old had the greatest rates of DALY (Fig. 2A), death (Fig. 2B), YLDs (Fig. 2C), and YLLs (Fig. 2D) in MENA and its countries in 2021. Also, the rates showed a decreasing trend with the advancement of age. Males had higher DALY (Fig. 2A), death (Fig. 2B), and YLL rates (Fig. 2D), while females showed greater YLD rates (Fig. 2C).

### Association with the socio-demographic index

**Fig. 3:**
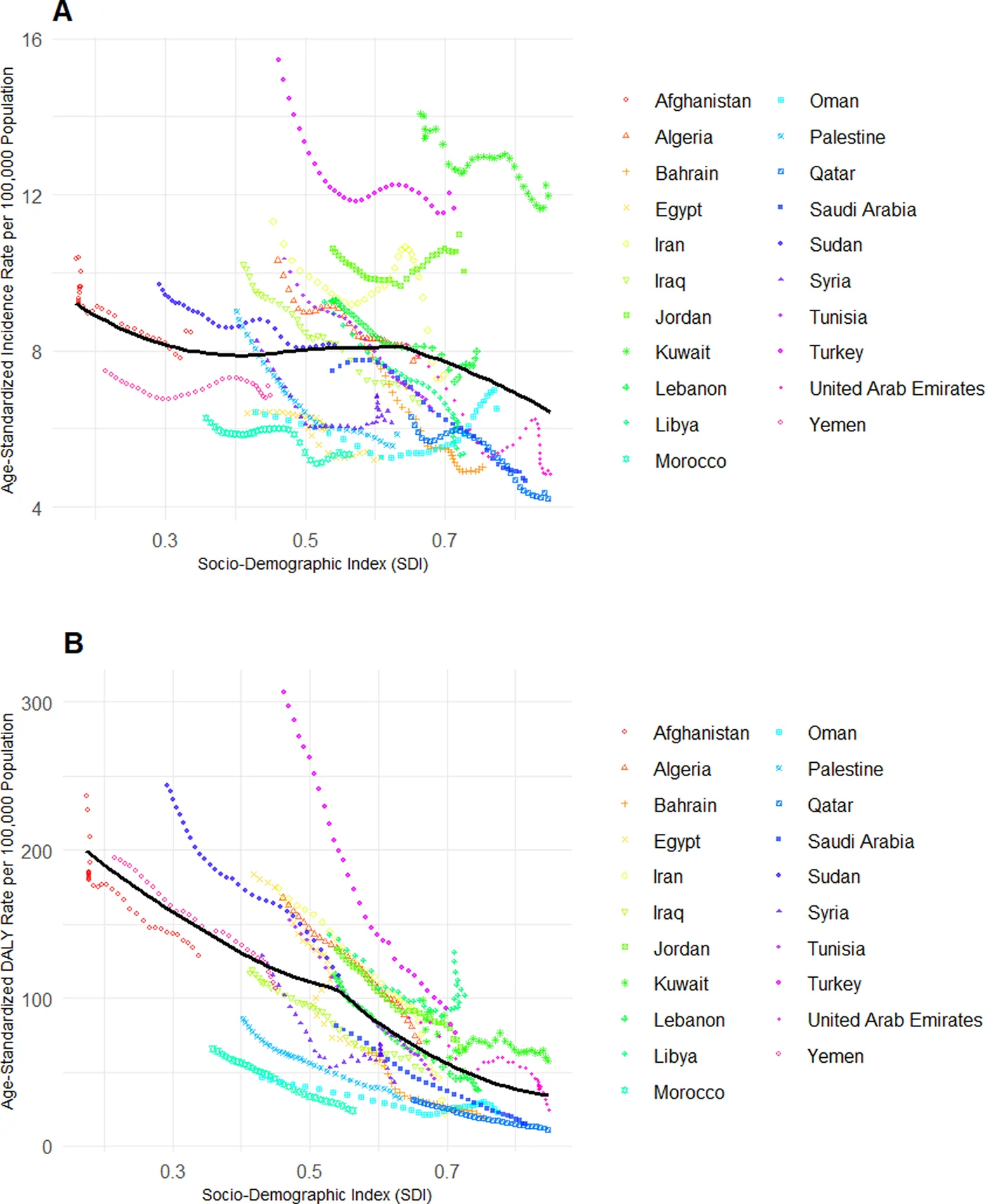
Age-standardized incidence rates (**A**) and age-standardized DALY rates (**B**) for digestive congenital anomalies per 100,000 population in countries of the Middle East and North Africa (MENA) region, categorized by SDI from 1990 to 2021. The expected values, based on SDI and disease rates across all locations, are represented by the black line. Each point represents a country’s observed age-standardized rate per 100,000 population from 1990 to 2021. DALY: disability-adjusted life year; SDI: Socio-demographic Index. (Generated from data available from http://ghdx.healthdata.org/gbd-resultstool)

The age-standardized incidence rates of DCAs decreased with SDI up to 0.4, then increased with SDI up to 0.6, then decreased after that. Countries like Kuwait, Jordan, and Türkiye had higher than expected burdens, while some other nations, such as Saudi Arabia, Yemen, Oman, Bahrain, and Egypt, showed lower than expected (Fig. 3A).

There was a negative association between age-standardized DALY rates of DCAs and SDI in MENA. Countries like Türkiye, Sudan, Kuwait, and Algeria had higher than expected burdens, while some other countries like Iraq, Morocco, Oman, Palestine, Qatar, Saudi Arabia, and Bahrain indicated lower than expected (Fig. 3B).

## Discussion

Our findings revealed that the global burden of DCAs has significantly reduced prevalence, DALYs, and death rates since 1990. In the MENA region, similar declines were observed, with decreases in incidence, mortality, and DALY rates. Kuwait had the highest incidence and prevalence rates at the national level, while Qatar reported the lowest. Afghanistan recorded the highest DALY and mortality rates, whereas Qatar had the lowest.

The analysis reveals significant differences in disease burden and mortality rates across various age groups and sexes. The results indicated that newborns aged 0 to 6 days experienced the highest rates of DALY, mortality, YLD, and YLL. These findings underscore the critical importance of infant health and the need for targeted health interventions during this vulnerable period [8]. Additionally, the observed decreasing trend in these metrics with advancing age suggests that, as age increases, the burden of disease and mortality significantly decreases. This decline may be attributed to gradual improvements in health conditions and access to effective healthcare services at older age [9].

Sex differences are also clearly evident in the data. Males face higher rates of DALY, mortality, and YLL, indicating a greater burden of mortality and morbidity in this group. Conversely, females exhibit higher rates of YLD. Because DCAs are congenital conditions, this sex difference in YLDs should not be interpreted as women spending more years living with disability; rather, it may reflect differences in survival, disease severity, diagnosis, or health-care access and utilization by sex. These disparities highlight the need for sex-specific health interventions and can inform the design of targeted health programs [10]. Furthermore, age and sex patterns reveal that males experienced higher DALY and mortality rates, while females had greater YLD rates. The association with the SDI indicated an inverse relationship with age-standardized DALY rates. These findings suggest that as SDI increases, DALY rates decrease, emphasizing the importance of understanding these relationships for developing effective health strategies [11].

The findings highlight the necessity for health policies tailored to the needs of vulnerable populations, particularly newborns and young children. A precise understanding of sex disparities in health outcomes and their relationship with SDI can guide effective interventions to reduce mortality and morbidity rates. These challenges require focused public health strategies to improve health metrics and enhance the quality of life in the MENA region [12, 13].

This study indicated that the age-standardized incidence rate has experienced a notable reduction compared to the 32 years, possibly due to advancements in public health and the management of these anomalies [2]. However, the relatively wide UIs around several percent-change estimates, particularly for DALYs and deaths, indicate that the exact magnitude of the decline should be interpreted cautiously. The direction of change is more robust than the precise size of the reduction. In the MENA region, the incidence and prevalence of cases have clearly declined, indicating improved access to healthcare services. Furthermore, mortality rates due to these anomalies have significantly decreased in this region, suggesting the success of effective health policies [14].

There were differences in the burden of DCAs among various countries. Specifically, countries like Kuwait and Afghanistan face more serious challenges and require special attention to healthcare infrastructure and treatment services [15]. In contrast, countries like Qatar have successfully reduced disease burden and mortality, which can serve as a model for other countries in the region. In this regard, targeted health policies and improvements in healthcare infrastructure are necessary for high-burden countries to achieve better outcomes. These efforts can help reduce anomalies, especially DCAs, and improve the quality of life for affected individuals [16, 17]. Also, Iran showed one of the largest relative reductions in DALY rate over the study period. Because percent change is calculated relative to the 1990 baseline, a high baseline rate can produce a large relative decline. This pattern may also reflect improvements in neonatal care, surgical management, and referral access over time. However, the wide UIs suggest that the magnitude of this decline should be interpreted cautiously.

Our results showed higher age-standardized rates of YLD and prevalence in MENA compared to the world, while MENA had lower rates for other metrics. In addition, the MENA region showed a greater reduction in all metrics than the global values between 1990 and 2021. Although the age-standardized incidence and mortality rates in MENA were comparable to the corresponding global estimates, the region demonstrated a higher age-standardized point prevalence rates. This pattern may reflect improved survival among affected children in several MENA countries due to advances in neonatal intensive care, pediatric surgery, and supportive management, resulting in a greater accumulation of living cases over time. Furthermore, differences in case ascertainment, registry completeness, coding practices, and reporting quality between MENA countries and other regions may also contribute to the relatively higher observed prevalence estimates. Because GBD estimates rely on multiple modeled data sources, regional differences in data availability and reporting systems may influence the balance between incidence, prevalence, and mortality estimates. In developed countries, significant advancements in preventive measures and medical technologies have improved capabilities for diagnosing and treating congenital anomalies, contributing to increased survival rates among affected children. However, in some developing countries, medical conditions are considerably weaker, resulting in notable disparities between the Global North and South. These differences are evident in the prevalence and mortality rates, patient prognosis, and quality of life [18–21].

Based on the results, countries in the MENA region showed significant differences in disease burden and mortality rates. For instance, Afghanistan, which has the highest disease burden and mortality compared to other countries, reflects serious challenges in public health. In contrast, Qatar, with the lowest disease burden and mortality rates, appears to have effectively utilized its healthcare infrastructure and implemented successful policies [22, 23]. Additionally, the United Arab Emirates has a lower disease burden than Kuwait, yet still faces challenges [24]. Afghanistan, with the highest YLD and mortality, indicates more severe health and social crises [25, 26]. Meanwhile, countries like Qatar and the United Arab Emirates, with the lowest YLLs, have significantly reduced these areas [24, 27]. Overall, these results highlight the need for special attention to health policy and infrastructure improvement in countries like Kuwait and Afghanistan to achieve better outcomes.

Our study showed that countries with lower socioeconomic status in the MENA region, determined by SDI, had higher age-standardized DALY and incidence rates. The findings are in accordance with the study by Liu and colleagues, who investigated the burden of different congenital anomalies in 2021 globally [28]. The increased burden in regions with lower SDI values can be due to several factors, including restricted access to prenatal care, poor maternal nutrition, and inadequate healthcare systems for early detection and management of these conditions [28]. Furthermore, many lower-income countries lack comprehensive birth defect surveillance systems, resulting in underreporting and delayed interventions [29]. To address these disparities, targeted public health initiatives are essential, such as strengthening prenatal screening efforts and improving healthcare accessibility in underserved areas. Implementing these strategies can help alleviate the burden of DCAs and reduce health inequalities across varying socioeconomic settings.

The findings of the study can assist health policymakers in the MENA region in designing more effective strategies to reduce the burden of DCAs. The significant reductions in incidence, prevalence, and DALY rates indicate the impact of previous interventions and can serve as a model for future planning. Additionally, identifying countries such as Afghanistan with the highest DALY rates and Qatar with the lowest can help in the targeted allocation of resources. The relationship between DCAs and SDI suggests that socioeconomic development plays an important role in shaping both the observed burden and the outcomes of affected children. The negative association between DALY rates and the SDI also highlights the importance of investing in social and economic development. This pattern may reflect limited access to high-quality antenatal screening, delayed diagnosis, shortages of neonatal intensive care and pediatric surgical services, and weaker referral systems in lower-SDI settings. In addition, underreporting and incomplete surveillance may lead to uncertainty in the recorded incidence in some countries, especially where health information systems are less developed. The pattern observed for incidence shows that in lower-SDI settings, weaker diagnostic systems may reduce case detection, whereas in middle- and higher-SDI settings, improved prenatal screening and better registration may increase the identification of cases. At the same time, better neonatal and surgical care may improve survival, which can increase prevalence while reducing mortality and DALYs. At the country level, the higher-than-expected burden may reflect differences in healthcare access, neonatal care capacity, and health-system performance, whereas lower-than-expected burden may be associated with stronger maternal-child health services and earlier diagnosis and treatment.

A strength of the study is that we have provided up-to-date and comprehensive estimates of levels and trends associated with DCAs between 1990 and 2021. Nonetheless, this study has limitations. First, the GBD estimates are model-based and depend on the availability, completeness, and quality of source data. Therefore, sparse vital registration, underreporting, misclassification, and coding inconsistencies may influence the estimates, particularly in countries with limited surveillance systems or disrupted health services. Second, because the GBD framework combines multiple data sources and applies statistical modeling and imputation, some country-level estimates may be accompanied by wide uncertainty intervals and may not fully account for local clinical realities. Third, the study is based on aggregated regional and national estimates, which limits our ability to assess subtype-specific DCAs or within-country variation. Fourth, the SDI is a broad composite measure and cannot account for important contextual determinants such as maternal nutrition, consanguinity, environmental exposures, prenatal diagnostic capacity, or the availability of pediatric surgical care. Therefore, caution is warranted when interpreting its results, with particular attention to the noted limitations. Further research is necessary to address these gaps and better understand the trends and factors influencing global diseases.

## Conclusions

The prevalence, mortality, and DALYs due to DCAs are generally declining, reflecting ongoing progress in the MENA region. DCAs present a complex and diverse issue that requires the attention and support of society as a whole. With advancements in technology and the formulation of public health policies through international collaboration, better treatment and management for these individuals can be provided, leading to improved the quality of life and reducing the burden.

## Data Availability

The data used for these analyses are all publicly available at https:/vizhub.healthdata.org/gbd-results.
